# Irradiation Induces Epithelial Cell Unjamming

**DOI:** 10.3389/fcell.2020.00021

**Published:** 2020-02-11

**Authors:** Michael J. O'Sullivan, Jennifer A. Mitchel, Amit Das, Stephan Koehler, Herbert Levine, Dapeng Bi, Zachary D. Nagel, Jin-Ah Park

**Affiliations:** ^1^Department of Environmental Health, Harvard T. H. Chan School of Public Health, Boston, MA, United States; ^2^Department of Physics, Northeastern University, Boston, MA, United States

**Keywords:** epithelium, irradiation, migration, unjamming, metastasis, cancer

## Abstract

The healthy and mature epithelial layer is ordinarily quiescent, non-migratory, solid-like, and jammed. However, in a variety of circumstances the layer transitions to a phase that is dynamic, migratory, fluid-like and unjammed. This has been demonstrated in the developing embryo, the developing avian airway, the epithelial layer reconstituted *in vitro* from asthmatic donors, wounding, and exposure to mechanical stress. Here we examine the extent to which ionizing radiation might similarly provoke epithelial layer unjamming. We exposed primary human bronchial epithelial (HBE) cells maintained in air-liquid interface (ALI) to sub-therapeutic doses (1 Gy) of ionizing radiation (IR). We first assessed: (1) DNA damage by measuring p-H2AX, (2) the integrity of the epithelial layer by measuring transepithelial electrical resistance (TEER), and (3) the extent of epithelial cell differentiation by detecting markers of differentiated airway epithelial cells. As expected, IR exposure induced DNA damage but, surprisingly, disrupted neither normal differentiation nor the integrity of the epithelial cell layer. We then measured cell shape and cellular migration to determine the extent of the unjamming transition (UJT). IR caused cell shape elongation and increased cellular motility, both of which are hallmarks of the UJT as previously confirmed. To understand the mechanism of IR-induced UJT, we inhibited TGF-β receptor activity, and found that migratory responses were attenuated. Together, these observations show that IR can provoke epithelial layer unjamming in a TGF-β receptor-dependent manner.

## Introduction

The healthy and mature epithelial layer is ordinarily quiescent, non-migratory, solid-like, and jammed. In this jammed layer, epithelial cells maintain cobblestone-like shapes and rarely rearrange with their neighbors (Bi et al., 2014; Park et al., 2015; Atia et al., 2018). However, in a variety of circumstances the layer transitions to a phase that is dynamic, migratory, fluid-like and unjammed. In the unjammed layer, epithelial cells become elongated in shape and rearrange cooperatively with their neighbors (Sadati et al., 2013; Fredberg, 2014; Park et al., 2015, 2016; Pegoraro et al., 2016; Atia et al., 2018). This phase transition from the jammed to the unjammed phase, called the unjamming transition (UJT), was discovered in a living system using well-differentiated human bronchial epithelial (HBE) cells exposed to mechanical compression that mimics the mechanical effect of bronchospasm (Park et al., 2015). Since this discovery of these dynamic and structural hallmarks of epithelial UJT (Park et al., 2015; Atia et al., 2018), work from our team and others now suggests that the UJT seems to be a normal and perhaps essential part of epithelial biology but, nonetheless, can become hijacked in disease. For example, in the healthy embryo *in vivo* the UJT is triggered during ventral furrow formation during gastrulation in *Drosophila* (Atia et al., 2018), during elongation of the vertebrate body axis in the embryonic zebrafish (Mongera et al., 2018), and during airway epithelial branching in the embryonic avian lung (Spurlin et al., 2019). The UJT is therefore observed across vastly diverse biological contexts, in normal development and disease, both *in vitro* and *in vivo*, but its mechanisms and stimuli provoking the UJT are unknown.

Radiation therapy is often used to kill or shrink tumors (Molina et al., 2008; Chaffer and Weinberg, 2011). However, a growing body of evidence indicates that radiation therapy sometimes results in increased cancer metastasis among surviving cells (Moncharmont et al., 2014; Vilalta et al., 2016). The mechanism underlying this paradoxical radiation-induced metastasis remains unidentified but may involve a fluidization of the healthy, non-cancerous stroma which normally restrains the tumor. Here, we hypothesize that this paradoxical result is due to the unwanted effect of radiation on epithelial unjamming and resulting cellular migration. To test this hypothesis, we used primary HBE cells as a well-established model of the jammed layer, which can undergo the UJT. To test if radiation can provoke the UJT, we exposed the jammed HBE cell-layer to ionizing radiation (IR).

## Results

### Ionizing Radiation Induces DNA Damage

**Figure 1:**
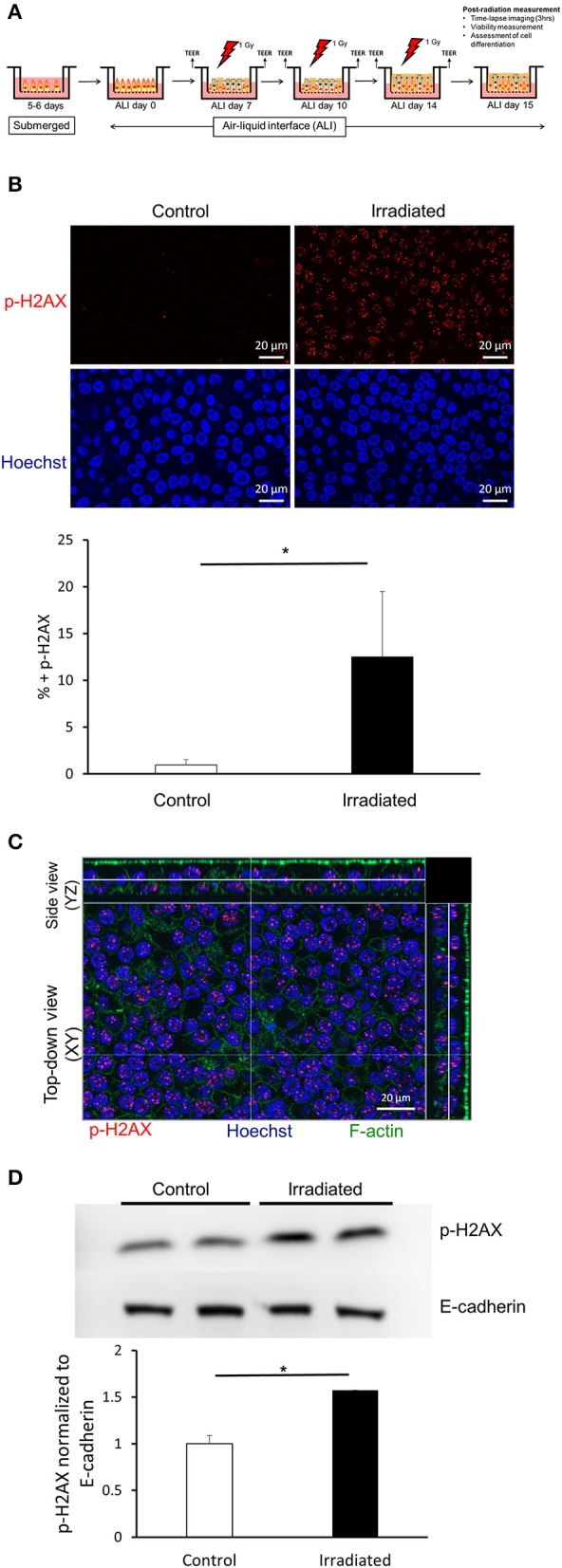
Ionizing radiation induces DNA damage. **(A)** Timeline of the experimental protocol performed to investigate epithelial cell unjamming induced by ionizing radiation. In primary HBE cells maintained in air-liquid interface culture exposed to ionizing radiation (IR), we determined DNA damage, barrier integrity, cellular viability, epithelial differentiation, as well as cellular shape and migration. **(B)** Representative images of p-H2AX (top, red) and nuclei stained with Hoechst (bottom, blue) from six independent experiments. IR exposure induced p-H2AX indicating increased DNA damage. Images were captured with a 63X objective (scale bars = 20 μm). The quantification of the mean area of positive p-H2AX staining is presented in the graph. Error bars represent the standard deviation from four FOVs (*n* = 4) from one representative experiment. **(C)** Representative orthogonal images of p-H2AX (red), F-actin (green), and nuclei stained with Hoechst (blue). Images were captured with a 63X objective (scale bar = 20 μm). **(D)** Western blotting confirms that IR induced p-H2AX. Quantified p-H2AX normalized to E-cadherin is presented in the graph. Error bars represent the standard deviation from two samples (*n* = 2).

Air-liquid interface (ALI) culture recapitulates the environment in which bronchial epithelial cells exist within the airway *in vivo*. Over 14 days in ALI, progenitor basal cells differentiate into a diverse population of epithelial cells as found *in vivo* (Fulcher et al., 2005). To assess DNA damage, we exposed cultures of primary HBE cells in ALI conditions to 1 Gy on ALI day 14. To determine the level of DNA damage, we performed immunofluorescent staining to detect p-H2AX, a marker for DNA-double strand breaks (DSBs) (Kuo and Yang, 2008). As previously reported in a different type of cells (Mariotti et al., 2013), we observed a maximal increase in p-H2AX at 1 h post-irradiation (data not shown). This maximal p-H2AX was reduced back to baseline by 6 h post-irradiation (data not shown). Compared to time-matched control cells, irradiated cells showed robust increases in the level of p-H2AX, indicating that exposure to IR indeed leads to DNA damage (Figure 1B). We observed positive p-H2AX in both apical and basolateral HBE cells as demonstrated by orthogonal side-view imaging (Figure 1C). We also observed increased p-H2AX protein by western blot (Figure 1D). Collectively, these data indicate that exposure of HBE cells to IR induces DNA damage.

### Ionizing Radiation Does Not Disrupt Normal Epithelial-Cell Functions

**Figure 2:**
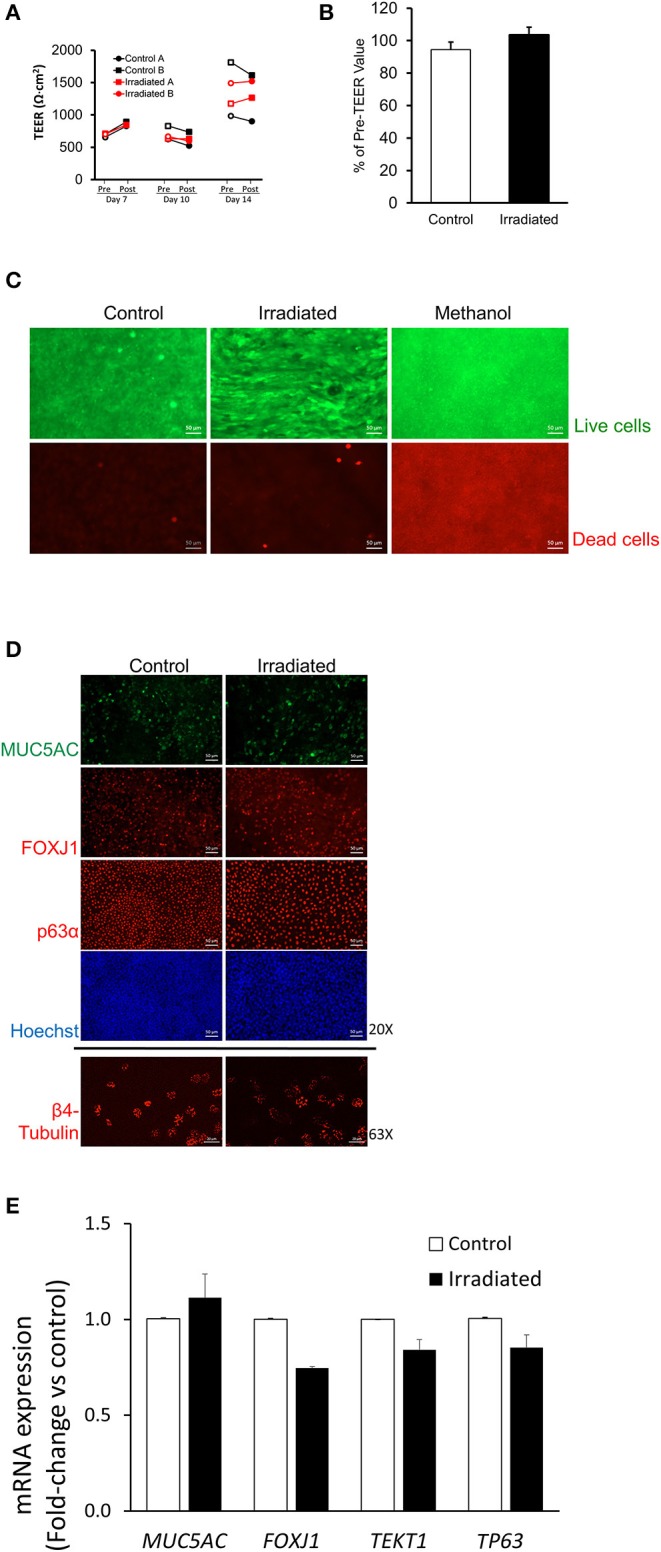
Ionizing radiation does not disrupt normal epithelial cell functions. **(A)** On ALI days 7, 10, and 14, TEER measured 1 h before (pre-exposure, open symbols) and 1 h after (post-exposure, closed symbols) indicates that IR exposure did not reduce TEER. Black lines represent TEER measured from two independent wells (Control A and Control B) of control and red lines represent TEER measured from two independent wells (Irradiated A and Irradiated B) of irradiated cells from one donor. **(B)** On ALI day 14, the percent change in TEER comparing 1 h pre-, and 1 h post-IR exposure showed no difference between control (white bar) and irradiated (black bar) cells, indicating that IR did not reduce TEER. Error bars represent the standard error of the mean from four independent donors (*n* = 4). **(C)** Methanol treatment used as a positive control for cell death resulted in prominent cell death as indicated by EthD-1 staining (red), whereas neither control nor IR exposure induced cell death (scale bars = 50 μm). Representative images **(D)** of MUC5AC, FOXJ1, p63α (20X), and β4-tubulin (63X). RT-qPCR **(E)** showing mRNA expression of *MUC5AC, FOXJ1, TEKT1*, and *TP63* indicate no differences in cellular differentiation between control and irradiated cells. Scale bar = 50 μm (top 8 panels), 20 μm (bottom 2 panels) in **(D)** and error bars represent the standard deviation from two representative donors (*n* = 2) in **(E)**.

To determine if DSBs induced by IR led to disruption of normal epithelial functions, we assessed barrier integrity, cellular viability, and epithelial differentiation. On ALI days 7, 10, and 14, barrier integrity of the HBE-cell layer was measured by transepithelial electrical resistance (TEER) at 1 h before and after each IR exposure (1 Gy) (Figure 2A). As expected for the normally maturing airway epithelial layer, TEER increased over ALI days, as previously reported (Park et al., 2015). In control HBE cells there was no difference in TEER before and after sham treatment. To our surprise, however, in irradiated cells there was also no difference in TEER before and after radiation exposure. Across four independent HBE-cell donors, TEER values post-irradiation on ALI day 14 showed no reduction compared with control (Figure 2B), thus indicating that repeated exposure to IR (1 Gy) did not compromise barrier function. At 24 h after the final exposure to radiation, we also examined cell viability by dead-cell staining with ethidium homodimer (EthD-1) that is only permeable to dead cells (Somodi and Guthoff, 1995). As a positive control for cell death, we treated cells with 70% methanol and detected robust reductions in cellular viability as expected. However, we observed no significant difference in cellular viability between the control and the irradiated cells, (Figure 2C) further indicating that repeated exposure to radiation (1 Gy) did not affect cellular viability.

During ALI culture conditions, basal HBE cells differentiate into goblet and ciliated cells (Stewart et al., 2012; Figure 2D). In control and irradiated cells, we observed a similar degree of differential populations of HBE cells by immunofluorescence staining for epithelial cell-type markers: MUC5AC for goblet cells; FOXJ1 and β4-tubulin for ciliated cells; p63α for basal cells (Figure 2D). Higher magnification, orthogonal side-view images of each cell marker shows that irradiated cells indeed differentiate, as exhibited by their expected localization in the baso-lateral plane (Supplementary Figure 1).

Quantification of the differential cell markers is technically challenging in the pseudostratified epithelium. Therefore, we analyzed mRNA expression of differentiated epithelial cell-type markers by RT-qPCR analysis (Figure 2E). We also measured mRNA expression of *TEKT1*, a gene encoding tektin-1 protein expressed in cilia (Yoshisue et al., 2004; Park and Tschumperlin, 2009). In control and irradiated cells, there was no difference in the levels of mRNA expression of *MUC5AC, FOXJ1, TEKT1*, and *TP63*. Collectively, these data demonstrate that repeated exposure of the primary HBE cells to radiation (1 Gy) induces DNA damage but does not affect epithelial layer integrity, cellular viability, or cellular differentiation.

### Ionizing Radiation Induces Epithelial Unjamming in a TGF-β Receptor-Dependent Manner

**Figure 3:**
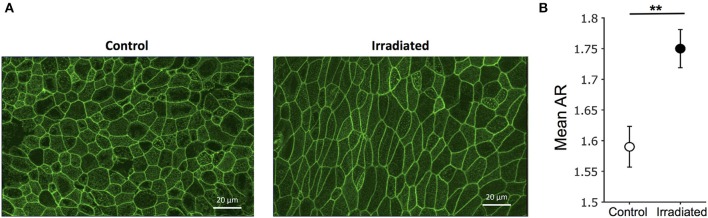
Ionizing radiation induces cell shape changes. **(A)** Representative images of F-actin staining indicate cobblestone-like shape in control (left) and elongated shape in irradiated cells (right) (scale bars = 20 μm). **(B)** Segmented images of F-actin staining (as shown in **A**) were used to calculate aspect ratio (AR = length of long axis/length of short axis). Mean aspect ratio, mean (AR), was calculated in four fields of view. Error bars represent the standard error of the mean from five independent experiments (*n* = 5).

To determine UJT in HBE cells, we measured cellular shape and motility. Because structural signatures reflect dynamic process of the UJT, we first used cell shape analysis to assess UJT (Park et al., 2015; Atia et al., 2018). We fixed cells at 24 h after the final exposure to IR and stained for F-actin to mark cell boundaries (Figure 3A). Control cells maintained a cobblestone-like shape whereas irradiated cells became elongated (Figure 3A). To determine systematic differences of cell shapes between control and irradiated cells, we measured cellular aspect ratio (AR) (Mashburn et al., 2012). Mean AR showed a significant difference between control and irradiated cells (Figure 3B), suggesting that irradiation induced cell shape elongation. In addition, we took time-lapse images of the pseudostratified epithelial layers starting at 5 h post-irradiation and analyzed cell motility and dynamics. Through optical flow analysis, we computed root-mean-square (RMS) displacements over 3 h. Compared to the time-matched controls, exposure to IR significantly increased the average RMS displacement of the HBE cells (Control: 0.79 ± 0.09 μm vs. IR: 6.21 ± 1.95 μm) (Supplementary Video 1).

**Figure 4:**
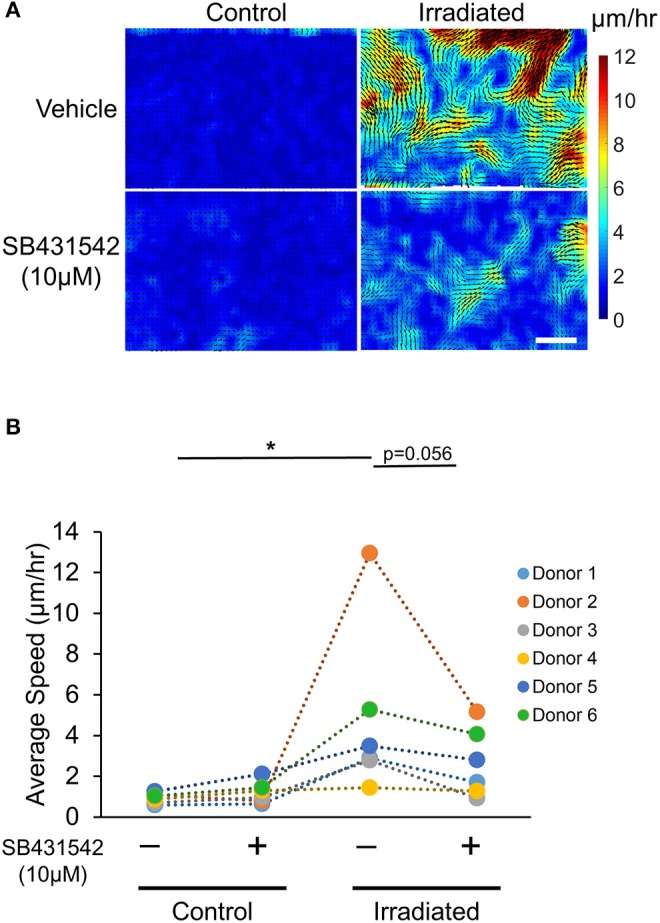
Irradiation-induced collective cell migration partially depends on TGF-β receptor. Representative displacement maps overlaid with velocity heat maps **(A)** and the quantified cellular motility **(B)** indicate that IR induced cellular motility, which was attenuated by SB431542 (a TGF-β receptor inhibitor) (Scale bar = 100 μm). Six donors (*n* = 6) were used as indicated.

To test whether inhibition of TGF-β receptor activity prevents the IR-induced UJT, HBE cells were pre-treated with the TGF-β receptor inhibitor, SB431542 (SB, 10 μM) (or vehicle) for 1 h prior to each IR exposure. Pre-treatment with the TGF-β receptor inhibitor partially attenuated the IR-increased average displacement of the cells (veh + IR: 6.21 ± 1.95 μm vs. SB + IR: 2.60 ± 0.75 μm; Figures 4A,B). Taken together, these data indicate that exposure of airway epithelial cells to IR provokes a UJT, accompanied by cell shape change. This IR-induced UJT is partially mediated via TGF-β receptor signaling. Because the activation of TGF-β receptor pathway is known to induce epithelial-to-mesenchymal transition (EMT) (Xu et al., 2009; Nieto et al., 2016), we examined if irradiated cells were undergoing EMT. While the cells treated with TGF-β1, as a positive control for the EMT, showed substantially increased expression of EMT marker genes, including *FN-EDA, VIM*, and *ZEB1*, irradiated cells were unchanged in their mRNA expressions (Supplementary Figure 2).

## Materials and Methods

### Culture of Primary Human Bronchial Epithelial Cells

Primary HBE cells were obtained from the CF Center Tissue Procurement and Cell Culture Core, the University of North Carolina at Chapel Hill (courtesy of Dr. Scott Randell). As previously described (Park et al., 2015), these cells were isolated from lungs unsuitable for transplant. The lungs were obtained under protocol #03-1396 approved by the Institutional Review Board at the University of North Carolina at Chapel Hill. Informed consent was obtained from authorized representatives of all organ donors. As previously described (Park et al., 2015; Mitchel et al., 2016), HBE cells were seeded on transwells coated with 0.05 mg/ml rat tail collagen 1 and maintained in ALI culture until the cells were well-differentiated as determined by immunofluorescent staining for goblet cells marked for MUC5AC, ciliated cells marked for FOXJ1 and b4-tubulin, and basal cells marked for p63α (Figure 2D).

### Exposure of Primary Airway Epithelial Cells to Ionizing Radiation

For the exposure of HBE cells to ionizing radiation, we used a RadSource RS 2000 Biological Research Irradiator (RadSource, Brentwood, TN). HBE cells were exposed to radiation over the course of ALI cultures, on days 7, 10, and 14. A dose of 1 Gy was achieved by setting the instrument to 160 kV, 25 mA for 87 s. During the radiation exposure, time-matched control (non-irradiated) cells were placed outside of the RadSource machine (Figure 1A demonstrates a timeline of the experimental procedures).

In experiments where TGF-β receptor was inhibited, SB431542 (10 μM) was added to the basolateral culture medium 1 h prior to each IR exposure. For vehicle of SB431542, DMSO (1%) was used.

### Immunofluorescent Staining

HBE cells on the transwell were fixed in 4% paraformaldehyde (PFA) for 30 min and subsequently washed with PBS. Cells were then permeabilized in 0.2% Triton X-100 in phosphate-buffered saline (PBST) for 10 min. Permeabilized cells were blocked in 10% non-specific goat serum and 1% bovine serum albumin (BSA) in PBST for 1 h. Primary antibody against p-H2AX (20E3, Cell Signaling Technology, Danvers, MA) was diluted at 1:200. Secondary antibody conjugated to Alexa Fluor 594 (ThermoFisher Scientific) was diluted at 1:500. Stained cells were mounted on coverslips and imaged with a fluorescent microscope (Axio Observer, Zeiss, Germany). To observe the differentiation of the ALI cultures, we stained fixed cells with primary antibodies for MUC5AC (45M1, ThermoFisher Scientific), FOXJ1 (2A5, ThermoFisher Scientific), β4-tubulin (ONS, Millipore Sigma), and p63α (D2K8X, Cell Signaling Technology). All primary antibodies were diluted at 1:500, followed by appropriate secondary antibody (AlexaFluor mouse/rabbit). Stained cells were counter stained for F-actin using phalloidin conjugated to AlexaFluor 488 (ThermoFisher Scientific) diluted at 1:40 and with Hoechst 33342 (ThermoFisher Scientific) diluted at 1:5000.

### Western Blotting

Protein lysates were separated in a 10% Mini-PROTEAN TGX pre-cast gel (Biorad). Proteins were transferred to a PVDF membrane which was subsequently blocked in 5% skim milk. The PVDF membrane was incubated with antibody against p-H2AX (20E3, Cell Signaling Technology) or E-cadherin (24E10, Cell Signaling Technology) and subsequently with secondary antibodies.

### RT-qPCR

RNA was extracted from cells using RNeasy Mini Kit (Qiagen, Valencia, CA) according to the manufacturer's instructions. One μg of total RNA was used to synthesize cDNA using MultiScribe reverse transcriptase (Applied Biosystems, Foster City, CA). RT-qPCR was performed using 25 ng of cDNA, primers (Table 1) and 2X SYBR Green PCR Mastermix.

**Table 1:** Primer sequences used in RT-qPCR.

**Genes**	**Primer sequences**	**References**
*GAPDH*	FW: 5′-TGGGCTACACTGAGCACCAG-3′ RV: 5′-GGGTGTCGCTGTTGAAGTCA-3′	Primer express 3 (Chu et al., 2006)
*MUC5AC*	FW: 5′-GGAACTGTGGGGACAGCTCTT-3′ RV: 5′-GTCACATTCCTCAGCGAGGTG-3′	Primer express 3
*FOXJ1*	FW: 5′-ATCCGCCACAACCTGTCTCT-3′ RV: 5′-CTTGCCTGGTTCGTCCTTCTC-3′	Primer express 3
*TEKT1*	FW: 5′-GCCCTTGCACATCACTGAGA-3′ RV: 5′-TCAATGCCAATGCGCTTCT-3′	Primer express 3
*TP63*	FW: 5′-GGACCAGCAGATTCAGAACGG-3′ RV: 5′-AGGACACGTCGAAACTGTGC-3′	Primer express 3
*FN-EDA*	FW: 5′-GAGCTATTCCCTGCACCTGATG-3′ RV: 5′-CGTGCAAGGCAACCACACT-3′	Doerner and Zuraw, 2009
*VIM*	FW: 5′-TGTCCAAATCGATGTGGATGTTTC-3′ RV: 5′-TTCTACCATTCTTCTGCCTCCTG-3′	Primer express 3
*ZEB1*	FW: 5′-GATGATGAATGCGAGTCAGATGC-3′ RV: 5′-ACAGCAGTGTCTTGTTGTTGT-3′	Tian et al., 2015

### Cell Shape Analysis

To determine if cell shape changes were affected by IR exposure, cells were stained for F-actin as described above. Then, maximum intensity projections of F-actin images were created in ImageJ software (Schindelin et al., 2012) and cells were segmented using SeedWaterSegmenter (Anaconda2). As previously reported (Atia et al., 2018), cellular aspect ratio (AR = long axis/short axis) was calculated and the mean AR was computed.

### Transepithelial Electrical Resistance

To determine if the integrity of airway epithelial cell layer is disrupted after radiation exposure, we used a widely accepted assay, TEER measurement, using an epithelial volt/ohm meter (EVOM2) (World Precision Instruments, Sarasota, FL), following the manufacturer's instruction, as we previously used (Park et al., 2015). In each well, TEER was measured 1 h before and 1 h after control treatment or irradiation. In some experiments, TEER was measured subsequently on ALI days 7, 10, and 14 (Figure 2A) following each exposure, whereas in other experiments (Figure 2B), TEER was measured only on ALI day 14 following the last exposure.

### Live/Dead Stain

To assess the viability of the cells post-radiation, cells were stained with calcein-AM and EthD-1 (Viability/Cytotoxicity kit, ThermoFisher Scientific, Waltham, MA) was performed according to manufacturer's protocol. As a positive control for cell death, other cells from independent culture wells were incubated with 70% methanol for 30 min. Briefly, at 24 h after the final IR, cells grown on transwells were incubated in PBS containing 2 μM calcein AM and 4 μM EthD-1 for 30 min. Then, stained cells were placed on glass coverslips and imaged at a single z-plane with a fluorescent microscope (Axio Observer, Zeiss, Germany).

### Time-Lapse Imaging

To monitor the motility of the cells after the final IR exposure, time-lapse images of the cells were taken at 3 min-intervals for 18 h. The imaging chamber was supplied with 37°C, 5% CO_2_ humidified air. A 10X objective, mounted on an Axio Observer (Zeiss, Germany) was used to collect phase contrast images. Each condition was run in duplicate and images from six fields of view per well were randomly collected. The average speed of cellular migration was assessed by Farneback optical flow analysis using Matlab (MathWorks, Natick, MA).

### Statistics

Statistical analysis was performed in GraphPad Prism 8 software (GraphPad Software Inc., San Diego, CA). Data are presented as mean + SD or + SEM as indicated, except Figure 3, which is represented by Mean ± SEM. In experiments with two groups, a two-tailed, paired Student's *t*-test was utilized. In experiments with more than two groups, a one-way ANOVA with Tukey's *post-hoc* test was utilized. *P*-values < 0.05 were considered to be significant.

## Discussion

The goal of this study is to provide the insight into the paradoxical effect of therapeutic radiation on cell motility (Moncharmont et al., 2014; Vilalta et al., 2016; Blyth et al., 2018). To address this question, we utilized *in vitro* culture of primary human airway epithelial cells as a model of the healthy, mature epithelium. In this epithelial cell layer system, irradiation caused DNA damage but did not disrupt normal differentiation or layer integrity. Irradiation did, however, induce cellular migration and cell shape elongation, both hallmarks of the UJT (Bi et al., 2015; Park et al., 2015; Atia et al., 2018). Our data therefore indicate that IR induced the UJT of well-differentiated, healthy HBE cells in a TGF-β receptor-dependent manner. This UJT of the non-cancerous healthy cells might create a fluidized environment, in which tumor cells might efficiently disseminate.

Under homeostatic conditions, the epithelium is typically non-migratory. However, under a variety of circumstances, the non-migratory layer becomes migratory. The transition occurs under both physiological circumstances, including wound healing, embryonic development, and pathophysiological circumstances, including tissue remodeling, cancer invasion, and metastasis. Transition of the non-migratory confluent epithelium toward the migratory state has traditionally been attributed to EMT or partial-EMT (pEMT) (Hay, 1982, 1995; Boyer et al., 1989; Savagner, 2015; Nieto et al., 2016; Brabletz et al., 2018), but in some cases can be attributed to the UJT (Sadati et al., 2013; Fredberg, 2014; Park et al., 2015, 2016; Pegoraro et al., 2016; Atia et al., 2018; Mitchel et al., 2019). The UJT together with associated fluidization of the confluent living tissue was first discovered in our laboratory (Trepat et al., 2009; Sadati et al., 2013; Park et al., 2015; Atia et al., 2018). This discovery was rooted firmly in airway biology. Since that time, the concept of cell jamming has diffused widely into the study of other collective cellular systems, and growing evidence from us and others now suggests that the capacity to jam and unjam may be an innate property of many epithelia both in normal development (Atia et al., 2018; Mongera et al., 2018; Spurlin et al., 2019) and in disease (Sadati et al., 2013; Haeger et al., 2014; Park et al., 2015; Bi et al., 2016; Gamboa Castro et al., 2016; Oswald et al., 2017; Atia et al., 2018; Mongera et al., 2018; Palamidessi et al., 2018, 2019; Fujii et al., 2019; Spurlin et al., 2019), including asthma (Angelini et al., 2011; Nnetu et al., 2012; Garcia et al., 2015; Park et al., 2015, 2016; Malinverno et al., 2017; Atia et al., 2018), and cancer (Haeger et al., 2014; Oswald et al., 2017; Palamidessi et al., 2019).

In cancer clinics, radiation is the most commonly used to kill or shrink tumors but sometimes results in the undesirable side effect of promoting migration and metastasis in surviving cancer cells. Further, tumor cells cultured in pre-irradiated stroma become highly metastatic, known as the tumor bed-effect (Monnier et al., 2008). However, these mechanism(s) remain unclear. In A549 cells (adenocarcinoma alveolar type II cells), IR (at 6 or 12 Gy) augments migration of the cells into a scratched-wound (Jung et al., 2007). This augmented migration is accompanied by cell-shape elongation (Jung et al., 2007). During cancer therapy, not only cancer cells, but also surrounding healthy epithelial cells are subjected to radiation. IR doses delivered to tumors often exceed a cumulative dose of 60 Gy, delivered over multiple treatment days (Kong et al., 2014). Delivery of high doses with minimal off-target damage is achieved by three-dimension-conformal treatment, which mitigates risk of exposure to nearby healthy tissue. Advances in radiotherapy delivery techniques have allowed for a sharp focus on the target tissue, limiting the damage to the surrounding stroma (Intensity Modulated Radiation Therapy Collaborative Working Group, 2001; Verellen et al., 2007). The resulting dose delivered to surrounding tissue is thus significantly lower than that which the tumor receives. Here, we utilized a 1 Gy dose, which is much lower than the therapeutic dose used in the clinic. Given three times over ALI culture of healthy primary HBE cells, the cumulative dose was 3 Gy. By the use of radiation at this dose, we did not observe cell-death but observed a striking migratory response, which could in turn affect physical behavior of neighboring cancer cells. Often, the radiated healthy epithelial cells are considered to promote cancer cell survival (Blyth et al., 2018). It has also been shown that low doses (0.8 Gy) of radiation may contribute to metastasis by increasing angiogenesis at the tumor site (Sofia Vala et al., 2010). However, the effect of radiation on cellular migration in healthy, mature, confluent airway epithelium had been unknown. Using primary HBE cells maintained in ALI, we examined the effects of irradiation on the homeostatic function of epithelial cells, as well as cellular migration.

Exposure of the cells to IR causes DNA damage by cleaving the phosphodiester bond in the backbone of the DNA (Lomax et al., 2013), leading to a DSB. Upon DSB, ATM Kinase phosphorylates H2AX followed by the cascade of DNA repair (Burma et al., 2001). Thus, we first determined if IR causes DSBs in HBE cells cultured at ALI. As we expected, exposure of HBE cells to IR (1 Gy) indeed caused DNA damage that is marked by p-H2AX (Figure 1C). Among the cellular events induced by IR, this observation can be particularly linked to asthma. In patients with asthma, the airway epithelium possesses an increased level of p-H2AX, suggesting that DNA damage is greater, or that DNA repair mechanisms are impaired in asthmatic airways (Chan et al., 2016). Furthermore, in a house dust mite (HDM) mouse model of allergic asthma, p-H2AX is increased in the airway epithelium (Chan et al., 2016). Surprisingly, a recent study suggests that IL-13 could be a therapeutic target for radiation-induced pulmonary fibrosis (Chung et al., 2016). IL-13 is a type 2 cytokine that is strongly associated with asthma (Rael and Lockey, 2011) and induces asthmatic airway remodeling, including increased subepithelial fibroblast proliferation (Kraft et al., 2001). Thus, there may be some common pathways that are shared between radiation-induced lung injury and asthma.

Despite substantial DNA damage marked by p-H2AX, radiation exposure at this sub-therapeutic dose (1 Gy) affected neither epithelial layer integrity nor normal epithelial differentiation, both of which are critical for maintaining proper epithelial functions (Figure 2). It is possible that radiation resulted in less p63α positive basal cells (Figure 2D), which may indicate the transition of basal into more differentiated cell types after exposure to DNA damage. However, we did not detect significant differences in *TP63* mRNA expression. In irradiated cells, we observed a small reduction in both marker genes for ciliated cells, *FOXJ1 and TEKT1* (Ryan et al., 2018), but it was not statistically significant (Figure 2E). Furthermore, we did not see any meaningful differences in any cell type marker proteins marked by immunofluorescent staining (Figure 2D), indicating that irradiated cells maintain their ability to differentiate similarly to non-radiated cells.

Without disruption of the epithelial barrier in the confluent epithelial layer, IR substantially induced cellular migration. To identify the mode of this increased cellular migration, we analyzed the cell shape and the speed of cellular motility to determine if this migration is attributable to the UJT. Although IR did not break cell-cell junctions as indicated by the lack of reduction in TEER (Figures 2A,B), IR caused significant changes in cell shape from cobblestone-like to elongated (Figure 3). IR also increased cellular motility (Figure 4), which was attenuated by pretreatment with a TGF-β receptor inhibitor. The TGF-β signaling pathway plays an important role in irradiation-induced fibrotic remodeling (Monson et al., 1998) and the inhibition of TGF-β signaling reduces irradiation-induced fibrosis in humans (Xavier et al., 2004). Furthermore, the increase in TGF-β expression in the mouse lung post-IR depends on IL-13, a type 2 cytokine (Chung et al., 2016), which is also tightly associated with asthma (Vignola et al., 1996; Minshall et al., 1997; Doran et al., 2017). TGF-β is a pleotropic factor that promotes a variety of asthmatic airway remodeling processes, including myofibroblast differentiation (Michalik et al., 2009), development of airway hyperresponsiveness (Leung et al., 2006), and airway smooth muscle proliferation (Makinde et al., 2007). Furthermore, TGF-β is a strong stimulator for epithelial cell migration through the EMT (Jakowlew, 2006; Al-Alawi et al., 2014; Nieto et al., 2016; Brabletz et al., 2018). In irradiated cells, our data indicate no signs of EMT (Supplementary Figure 2) but strong signs of UJT.

## Conclusion

In the present study, we investigated the unjamming transition, an emergent phenomenon in epithelial cell migration, induced by radiation exposure. Despite substantial DNA damage detected in the radiated cells, the integrity of the epithelial-cell layer, cellular viability, and degree of epithelial differentiation were not disrupted by radiation. Radiation induced the UJT that is characterized by increased collective cellular migration and elongated cell shape changes. Furthermore, blocking of TGF-β receptor attenuated the degree of radiation-induced cell migration, indicating a role for TGF-β receptor signaling in HBE-cell unjamming.

## Data Availability Statement

The datasets generated for this study are available on request to the corresponding author.

## Author Contributions

MO'S, JM, ZN, and J-AP designed the experiments. MO'S and JM performed experiments. MO'S, JM, AD, and SK analyzed the results. MO'S, JM, AD, SK, HL, DB, ZN, and J-AP interpreted the data and revised the manuscript. MO'S, JM, and J-AP drafted the manuscript.

### Conflict of Interest

The authors declare that the research was conducted in the absence of any commercial or financial relationships that could be construed as a potential conflict of interest.

## Supplementary Material

Supplementary Video 1 In control (left) and irradiated (right) cells, time-lapse images were taken between 5 and 8 h after exposure at an acquisition rate of every 3 min. In the converted movies, images are shown at 20 frames per second (one second of video is equal to 1 h of experimental time).

Supplementary Figure 1 Representative orthogonal (top-down view: X-Y and side view: Y-Z) and single z-plane (center) images from irradiated cells as shown in Figure 2D: MUC5AC (green), FOXJ1 (red), β4-tubulin (red), p63α (red), and Hoechst (blue). White line through orthogonal section indicates z-plane displayed (scale bar = 20 μm).

Supplementary Figure 2 In primary HBE cells, radiation did not induce EMT. To determine EMT, we measured mRNA expressions of EMT-related proteins, including fibronectin-EDA, vimentin and Zeb1 by RT-qPCR. In the cells exposed to TGFβ (10 ng/ml) as a positive control for the EMT, we detected a significantly increased expression of three genes, whereas in the cells exposed to radiation, we detected no meaningful increase in three genes, suggesting no sign of EMT.
