# Supplementary figures and images for: Irradiation Induces Epithelial Cell Unjamming
Source: Front Cell Dev Biol. 2020 Feb 11;8:21. doi: 10.3389/fcell.2020.00021 (PMC7026004; doi:10.3389/fcell.2020.00021)

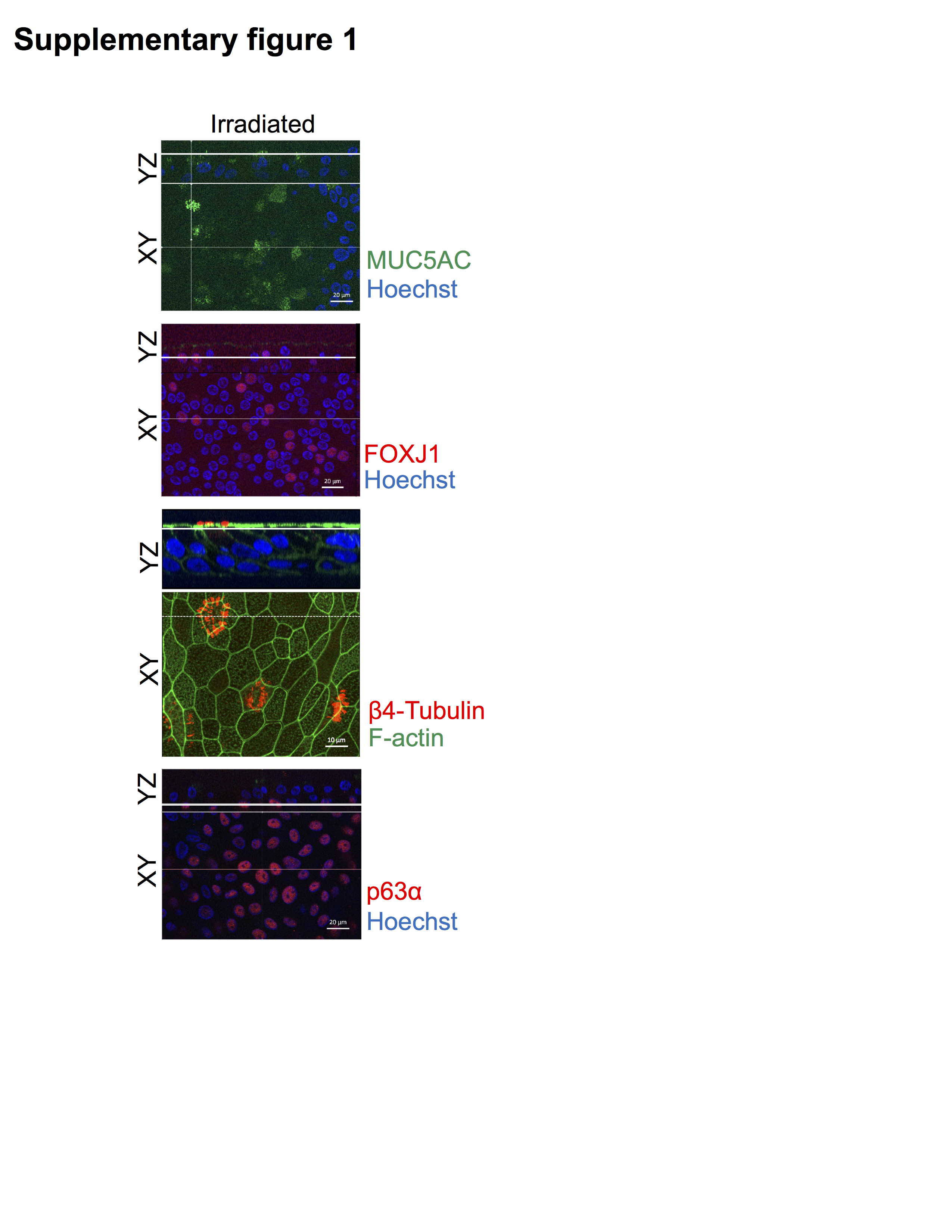

Supplement: Supplementary Figure 1 — Representative orthogonal (top-down view: X-Y and side view: Y-Z) and single z-plane (center) images from irradiated cells as shown in Figure 2D: MUC5AC (green), FOXJ1 (red), β4-tubulin (red), p63α (red), and Hoechst (blue). White line through orthogonal section indicates z-plane displayed (scale bar = 20 μm). [file Image_1.tiff]

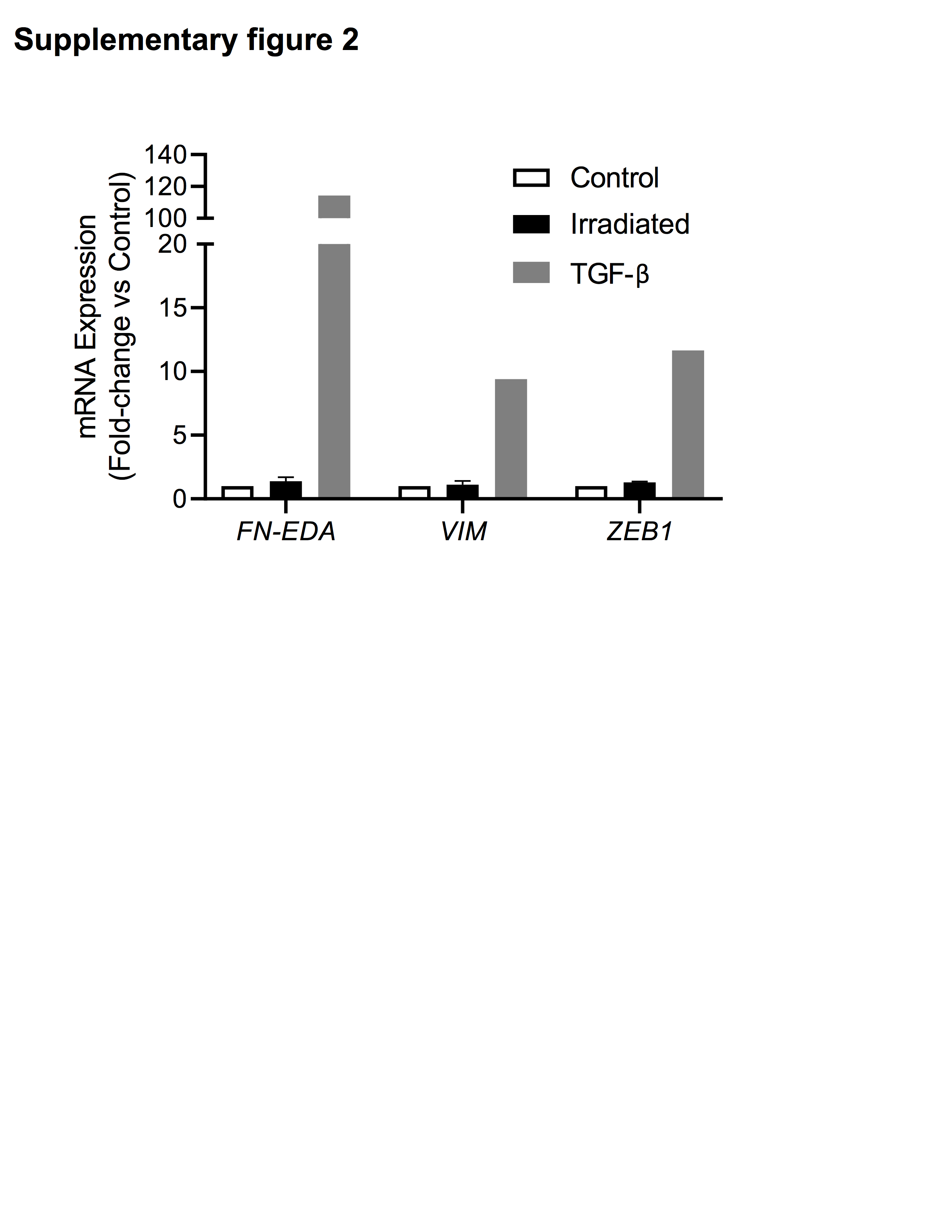

Supplement: Supplementary Figure 2 — In primary HBE cells, radiation did not induce EMT. To determine EMT, we measured mRNA expressions of EMT-related proteins, including fibronectin-EDA, vimentin and Zeb1 by RT-qPCR. In the cells exposed to TGFβ (10 ng/ml) as a positive control for the EMT, we detected a significantly increased expression of three genes, whereas in the cells exposed to radiation, we detected no meaningful increase in three genes, suggesting no sign of EMT. [file Image_2.tiff]
